# The role of quantitative deep capillary plexus in the pathogenesis of type 3 macular neovascularization: an optical coherence tomography angiography study

**DOI:** 10.1007/s00417-021-05330-w

**Published:** 2021-08-05

**Authors:** Marina Concilio, Federica Fossataro, Daniela Montorio, Mariapaola Giordano, Gilda Cennamo

**Affiliations:** 1 Eye Clinic, Department of Neurosciences, Reproductive Sciences and Dentistry, University of Naples Federico II, Via S. Pansini 5, 80131 Naples, Italy; 2 Eye Clinic, Department of Public Health, Federico II University, Naples, Italy

**Keywords:** Type 3 MNV, Optical coherence tomography angiography, Deep capillary plexus, Vessel density, Reticular pseudodrusen

## Abstract

**Purpose:**

To quantitatively investigate the role of deep capillary plexus (DCP) in patients affected by type 3 macular neovascularization (MNV), compared to patients with reticular pseudodrusen (RPD) eyes and healthy controls, using optical coherence tomography angiography (OCTA).

**Methods:**

In this prospective observational study, a total of seventy-eight eyes of 78 patients were included. Group 1 consisted of 40 eyes of 40 patients with stage 1 of type 3 MNV (22 males, 18 females, mean age 73.7, SD ± 6.60) and group 2 included 38 eyes of 38 patients with RPD (17 males, 21 females, mean age 73.2, SD ± 4.55). The control group included 40 eyes of 40 healthy subjects (20 males, 20 females, mean age 71.4, SD ± 6.36 years). We evaluated the retinal vessel density (VD) of superficial capillary plexus (SCP) and deep capillary plexus (DCP) using OCTA.

**Results:**

Patients with diagnosis of type 3 MNV showed statistically lower values of VD in DCP with respect to controls and to RPD group (*p* < 0.001), while there were no statistical differences between RPD and control group in macular region. No significant differences in VD of SCP were detected among the three study groups.

**Conclusion:**

OCTA provides a reproducible, non-invasive detailed quantitative analysis of retinal vascular features and changing in early-stage type 3 MNV patients, which allowed to shed the light on the main role of DCP ischemia in the development of type 3 MNV.

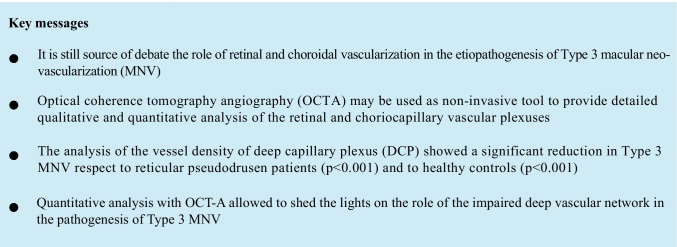

## Introduction

Retinal vascular anomalous complex was first described by Hartnett et al. [1] in 1992 as a distinct form of neovascularization (NV) in patients with age-related macular degeneration (AMD). Several hypotheses concerning the origin of this lesion have been postulated. Yannuzzi et al. [2] introduced the term *retinal angiomatous proliferation* (RAP), proposing an intraretinal origin of the NV. Thus, the authors described an abnormal proliferation of vessels in the middle and inner retina, which spread into the sub-retinal space and eventually communicated with choroidal vessels—the so-called retinal-choroidal anastomosis (RCA) [2]. Conversely, Gass et al. [3] in 2003 hypothesized a choroidal origin and referred to these vascular anomalous complex as *occult* *chorioretinal* *anastomosis*. The term type 3 NV, subsequently introduced by Freund et al. [4], expanded the Gass choroidal neovascularization (NVC) classification system omitting its origin.

In the last years, several studies performed to understand the pathogenesis of type 3 MNV, supported the intraretinal origin, highlighted early stages’ features of type 3 NV, and demonstrated the presence of reticular pseudodrusen as a risk factor to the development of type 3 MNV [5–9].

Furthermore, the introduction of optical coherence tomography angiography (OCTA), a novel and non-invasive tool, provided a detailed qualitative and quantitative analysis of the retinal and choroidal vascular plexuses, due to depth-resolved capability, allowing to clarify the type 3 MNV origin.

The aim of this prospective observational study was to quantitatively evaluate deep capillary plexus (DCP) in the early stage of type 3 MNV eyes compared to reticular pseudodrusen (RPD) eyes and healthy controls to better understand the pathophysiologic mechanism of this neovascularization.

## Materials and methods

In this prospective observational study, we recruited seventy-eight eyes of 78 AMD consecutive naïve patients with a diagnosis of type 3 MNV and RPD examined in the Eye Clinic of the University of Naples “Federico II” between September 2019 and December 2020.

The patient group included 40 AMD patients (40 eyes) with stage 1 of type 3 MNV, according to Su classification [10], and 38 patients (38 eyes) with RPD without drusen and outer retinal atrophy.

The control group consisted of forty eyes of 40 age-matched healthy subjects (20 males, 20 females, mean age 71.4, SD ± 6.36 years) with normal ophthalmological examination without ocular and systemic diseases.

Exclusion criteria were previous choroidal neovascularization secondary to other causes, previous anti-VEGF treatments, retinal vascular diseases, myopia greater than 6 diopters, history of ocular surgery, and significant lens opacity.

All the subjects underwent a complete ophthalmological examination including the measurement of best-corrected visual acuity (BCVA), according to the Early Treatment of Diabetic Retinopathy Study (ETDRS), slit-lamp biomicroscopy, fundus examination, multimodal imaging, namely multicolor imaging, infra-red (IR), fundus autofluorescence (FAF), fluorescein angiography (FA), indocyanine green angiography (ICGA), spectral-domain (SD)–optical coherence tomography (OCT) (Spectralis, Heidelberg Engineering, Heidelberg, Germany), and OCTA (AngioVue, RTVue XR Avanti, Optovue, Inc., Freemont, CA). All observations and the OCTA measurements were performed by two masked examiners (MC, DM) and a senior expert (GC) that confirmed the diagnosis of stage 1 type 3 MNV.

The study was approved by the Institutional Review Board of the University of Naples “Federico II” and all investigations adhered to the tenets of the Declaration of Helsinki. Written informed consents were obtained from the patients enrolled in the study. The research protocol was registered on clinical Trials.gov (protocol number: 2912/20).

### Optical coherence tomography angiography

OCTA images with the Optovue Angiovue System (software ReVue XR version 2018.1.1.60, Optovue Inc., Fremont, CA, USA) followed a standardized protocol based on the split-spectrum amplitude-decorrelation algorithm (SSADA), as previously described [11].

A 6 mm × 6 mm macular scan was performed to visualize the retinal capillary plexus in the macular region divided, according to the ETDRS classification of diabetic retinopathy, in the whole image, fovea, and parafovea.

The AngioAnalytic™ software automatically calculated in the two retinal vascular networks: superficial capillary plexus (SCP) and DCP, the vessel density (VD), defined as the percentage area occupied by the microvasculature in whole scan area and in all sections [12].

The software includes the 3D Projection Artifact Removal algorithm to improve the quality of OCTA images.

From the analysis were excluded the images with a signal strength index less than 60 and residual motion artefacts, incorrect segmentation, low centration, and focus.

### Statistical analysis

Statistical analysis was performed with the Statistical Package for Social Sciences (Version 25 for Windows; SPSS Inc, Chicago, IL, USA). One-way analysis of variance (ANOVA) followed by Bonferroni post hoc analysis was used to compare OCTA parameters among controls, type 3 MNV, and RPD groups. A *p* value of < 0.05 was considered statistically significant.

## Results

A total of seventy-eight eyes of 78 patients including 40 AMD patients with type 3 MNV (22 males, 18 females, mean age 73.7, SD ± 6.60) and 38 RPD patients (17 males, 21 females, mean age 73.2, SD ± 4.55) was enrolled in this study.

The age and sex did not significantly differ among the study groups (*p* = 0.191, *p* = 0.663; respectively).

BCVA was significantly impaired in type 3 MNV patients with respect to other groups (*p* < 0.001) while it did not differ between RPD patients and controls (Table 1).

**Table 1:** Demographic, ophthalmological characteristics of controls, RPD, and type 3 MNV patients

	Control group	RPD	Type 3 MNV	ANOVA
				*p*
Eyes (n.)	40	38	40	-
Gender (male/female)	20/20	17/21	22/18	0.663†
Age (years)	71.4 ± 6.36	73.2 ± 4.55	73.7 ± 6.60	0.191
BCVA (LogMAR)	0.16 ± 0.07	0.19 ± 0.07	0.35 ± 0.07	< 0.001

Data are expressed as mean ± SD

*RPD* reticular pseudodrusen; *MNV* macular neovascularization

*BCVA* best-corrected visual acuity; *logMAR* logarithm of the minimum angle of resolution

One-way analysis of variance (ANOVA) followed by Bonferroni post hoc analysis

^†^Chi-squared test, statistical significance *P* value < 0.05

Statistical significance *P* value < 0.05

OCTA examination showed no statistically significant difference in VD of SCP among the three study groups in the macular region.

**Fig. 1:**
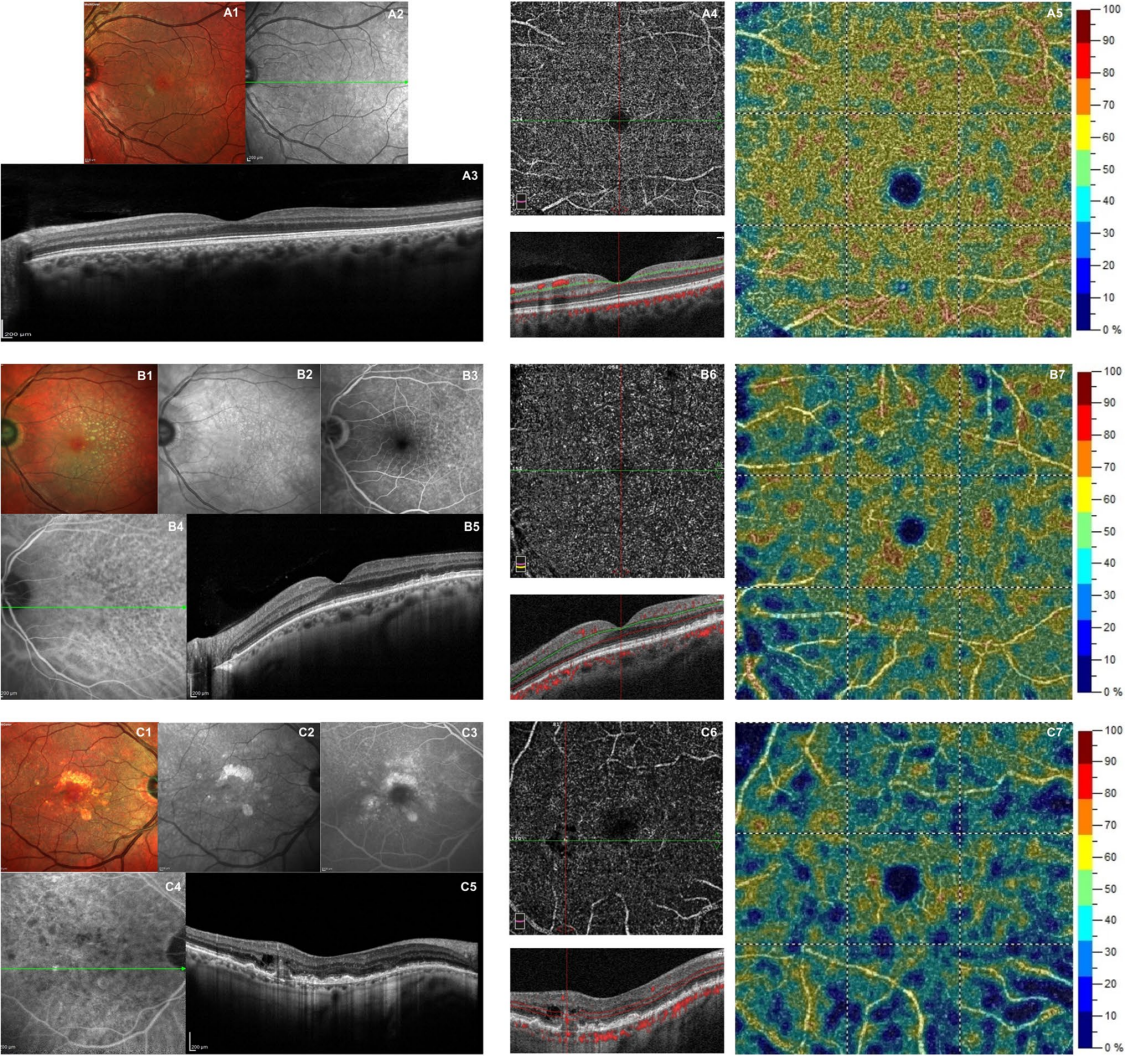
Top row. Left eye of a healthy subject (68-year-old female) shows a normal multicolor (A1) and infra-red imaging (A2), absence of structural retinal changes at spectral-domain optical coherence tomography (SD-OCT) B-scan (A3), normal morphology of deep capillary plexus (DCP) (A4), and normal vessel density of the DCP (A5) at OCT angiography (OCTA). Middle row. Left eye of a patient (65-year-old male) with reticular pseudodrusen reveals a yellowish-green reticular pattern at multicolor imaging (B1), doughnut pattern at infra-red imaging (B2), hyperreflective points at fluorescein angiography (B3), hyporeflective circular pattern at indocyanine green angiography (B4), and multiple accumulation of hyperreflective material above the retinal pigment epithelium (B5) at structural SD-OCT B-scan. OCTA images of the DCP (B6) present a focal reduction of vessel density in DCP (B7) respect to healthy subject. Bottom row. Right eye of a patient (64-year-old female) with diagnosis of type 3 MNV in stage 1 reveals yellowish-green reticular pattern, large yellowish drusen, and hemorrhage in macular region at multicolor imaging (C1), hyperreflective pattern and hyporeflective point corresponding to the hemorrhage at infra-red imaging (C2), and an area of hyperfluorescence at fluorescein angiography (C3). Indocyanine green angiography shows a focal area of hyperfluorescence or so-called “hot spot” representing the type 3 neovascularization (C4). SD-OCT shows corresponding reflectance within the retina at the level of outer and inner nuclear layers overlying a reticular pseudodrusen, with development of intraretinal fluid (C5). OCTA images reveals a tuft-like network with dark halo at the level of DCP with corresponding flow signal at the structural OCT B-scan (C6). A widespread reduction of vessel density of DCP was detected at OCTA respect to eyes with reticular pseudodrusen and healthy subjects (C7)

Different results were found in DPC analysis that revealed statistically lower values of VD in type 3 MNV patients with respect to controls (*p* < 0.001) while there were no statistical differences between the RPD group and controls in each macular sector (Fig. 1).

Comparing the two study groups, the VD of DCP was significantly decreased in type 3 MNV with respect to RPD patients (whole image *p* < 0.001; parafovea *p* < 0.001; fovea *p* < 0.001) (Table 2).

**Table 2:** Comparison of retinal vessel density among controls, RPD, and type 3 MNV patients

	Control group	RPD	Control vs RPD	Type 3 MNV	Control vs type 3 MNV	RPD vs type 3 MNV	ANOVA
							*p*
SCP (%)							
Whole image	46.76 ± 8.32	43.98 ± 7.18	0.375	42.90 ± 8.16	0.094	0.852	0.085
Parafovea	47.87 ± 7.07	45.85 ± 8.57	0.687	45.28 ± 6.29	0.354	1	0.260
Fovea	29.90 ± 6.65	27.91 ± 6.97	0.697	26.53 ± 8.15	0.123	0.921	0.120
DCP (%)							
Whole image	49.32 ± 7.43	46.91 ± 7.92	0.426	42.12 ± 6.17	< 0.001	0.012	< 0.001
Parafovea	51.31 ± 7.34	48.12 ± 8.59	0.222	43.58 ± 7.49	< 0.001	0.035	< 0.001
Fovea	41.64 ± 7.39	38.24 ± 7.24	0.170	33.28 ± 8.61	< 0.001	0.017	< 0.001

Data are expressed as mean ± SD

*RPD* reticular pseudodrusen; *MNV* macular neovascularization

*SCP* superficial capillary plexus; *DCP* deep capillary plexus

One-way analysis of variance (ANOVA) followed by Bonferroni post hoc analysis

Statistical significance *P* value < 0.05

## Discussion

To the best of our knowledge, no previous studies have been published about the quantitative analysis of DCP in the early stage of type 3 MNV, with OCTA. However, several reports analyzed in detail the microvascular morphology of type 3 MNV supporting the origin from the DCP and reporting the choroidal anomalies associated with this type of NV using OCT and OCTA [13–16].

A distinct tuft-like network, with high flow, origins at the DCP which could later progress towards the sub-retinal pigment epithelium (RPE) space, the RPE, and the choroid, causing exudation and pigment epithelial detachment, was described [10, 17, 18].

Consequently, different plexuses could get involved during type 3 MNV progression.

Sacconi et al. [5] first described the characteristics of early intraretinal NV originating from DCP, as the presence of intraretinal hyperreflective foci on structural OCT, corresponding to a detectable flow in DCP on OCTA in nascent type 3 NV. In both the preclinical stage and stage 1, the retinal vascular proliferation does not show a visible connection to the RPE and sub-RPE space or the choroid [5, 10]. Therefore, in the early stages of type 3 MNV, the DCP could be the first plexus involved.

In our prospective observational study, we evaluated VD of DCP in type 3 MNV eyes with stage 1 versus RPD eyes and healthy controls, using OCTA, to better understand the role of DPC in the pathogenesis of this type of NV.

Overall, we found a lower perfusion of the inner retina in the type 3 MNV group compared with RPD patients, while a mild decrease of VD, although not statically significant, was found in the RPD group versus controls.

Martins et al. [6] also noticed a significantly lower vascular perfusion of the DCP in patients with RAP.

Furthermore, in support of a retinal origin of type 3 MNV, several histopathologic studies demonstrated the evidence of intraretinal NV without the presence of sub-RPE vascular involvement [19, 20]. Therefore, it is plausible to hypothesize that an alteration of the DCP, specifically a reduction in VD, may favor the subsequent development of type 3 MNV. Retinal ischemia, due to a reduction in blood flow, with the consequent increase in VEGF could represent the *primum movens* of hypoxia [21].

Moreover, the changes of DCP were highlighted by Colantuono et al. [22] who suggested that, in different age-related degeneration (AMD) stages, alterations in both outer retina and RPE, which represent the main metabolic consumer in the retina, may cause an increase of oxygen from the choroid to the inner retina and, therefore, a vasoconstriction, visualized with a decrease of retinal perfusion in DCP on OCTA. The authors, indeed found a reduction of DCP in eyes with quiescent macular NV and intermediate AMD, while they report that in exudative AMD eyes, the perfusion density remained stable throughout follow-up, suggesting that anti-vascular endothelial growth factor (VEGF) injections do not impact on the perfusion of the DCP [22].

On the other hand, Borrelli et al. [23] showed that morphology of retinal capillary plexus is strongly linked to the metabolic demand of neuroretina and to its thickness, especially for DCP perfusion, demonstrating a direct relation between these two variables.

Therefore, a reduced perfusion of the inner retina could suggest that the early changes in DCP might represent a possible biomarker of type 3 MNV formation and our results could hence confirm the primary role of the DCP in the pathogenesis of type 3 MNV.

Furthermore, we also found a mild decrease in VD of DCP, although not statically significant, in the RPD group versus controls. Moreover, previous studies showed a reduction of choriocapillaris VD in RPD, as well as in eyes with type 3 MNV suggesting that this hypoperfusion could represent a hypoxic precursor for the onset of this MNV [13, 24, 25]. Therefore, the reduction of retinal and choriocapillaris VD may suggest a common origin of type 3 MNV and RPD and raise further interesting questions about the possible role of RPD as the precursor of type 3 MNV.

Future longitudinal studies on larger cohorts may more precisely evaluate the variations of DCP in the different stages of type 3 MNV, its causative role in the development and progression of this type of NV, and the possible correlation with the role of both choriocapillaris and choroid. This study had several limitations: firstly, the small sample size. Secondly, one of the most notable limitations of OCTA is image artefacts, due to distorted retinal structures and projection effects, which more profoundly impact on DCP evaluation rather than SCP and which we overcome using projection-resolved software.

In conclusion, our finding could shed the light on the role of DCP in the pathogenesis of type 3 MNV due to a detailed quantitative analysis of retinal vascular features provided by OCTA.
